# Parental perceptions of children’s exposure to tobacco smoke: development and validation of a new measure

**DOI:** 10.1186/s12889-018-5928-1

**Published:** 2018-08-20

**Authors:** Vicki Myers, Shoshana Shiloh, Laura Rosen

**Affiliations:** 1 Department of Health Promotion, School of Public Health, Sackler Faculty of Medicine, Tel Aviv University, Ramat Aviv, Israel; 2 School of Psychological Sciences, Tel Aviv University, Ramat Aviv, Israel

**Keywords:** Tobacco smoke exposure, Children, Parents, Perceptions, Secondhand smoke, Validation study

## Abstract

**Background:**

It is estimated that around 40% of children worldwide are exposed to tobacco smoke, largely by their parents. Discrepancies between biochemical measures of exposure and parental report imply that parents may be under-reporting children’s exposure. Previous research has shown that there may be a fundamental misunderstanding among smoking parents as to what exactly exposure is and in what circumstances it occurs.

**Methods:**

We aimed to develop and validate a measure to assess parental perceptions of exposure (PPE) regarding child tobacco smoke exposure (TSE). A model was developed based on a qualitative study of smoking parents and a questionnaire constructed using pictures and vignettes to assess parental rating of children’s exposure in hypothetical situations. The questionnaire was completed online by 220 Israeli parents recruited via social media. Exploratory factor analysis was performed, and reliability and internal consistency were assessed using test-retest reliability and Cronbach’s alpha coefficient.

**Results:**

Factor analysis produced 6 factors for PPE which explained a cumulative total variance of 76.3%. Factors were termed: 1) second-hand exposure; 2) third-hand exposure; 3) perceived knowledge/certainty; 4) sensory perceptions; 5) time perceptions; and 6) distance perceptions. All sub-scales showed good internal consistency and variance. Test-retest reliability was high (*r* = 0.856, *p* = .001). Total PPE score and subscales were highly correlated with risk perceptions *r* = 0.766. Smokers scored significantly lower on PPE than non-smokers, defining fewer situations as involving greater exposure (*p* < 0.001). Logistic regression showed PPE was able to discriminate smoking status.

**Conclusions:**

Results provide supporting evidence for the PPE as a reliable and valid construct, which can be feasibly measured. Smokers perceived exposure less frequently than non-smokers. This new measure can shed light on parental smoking behaviour and may help us to increase parental awareness of exposure in order to potentially reduce children’s exposure to tobacco smoke.

**Electronic supplementary material:**

The online version of this article (10.1186/s12889-018-5928-1) contains supplementary material, which is available to authorized users.

## What this study adds

There may be a fundamental misunderstanding among smoking parents regarding when exposure to smoking occurs. We repeatedly see that parents have diverse interpretations of the standard questions asked by researchers to determine children’s exposure. We designed and validated the Parental Perceptions of Exposure tool to identify and quantify parents’ perceptions of children’s exposure, with the long-term aim of changing these perceptions in order to reduce children’s exposure. Our findings demonstrated that parental perceptions of exposure can be reliably quantified; that smoking parents perceive exposure less frequently than non-smoking parents; and that PPE can discriminate smoking status.

## Background

A large proportion of children worldwide are exposed to tobacco smoke, often in the home [1, 2]. While adult smokers have some choice regarding whether or not to smoke, children who live, play and study in areas frequented by smokers are forcibly exposed to the harms of second-hand smoke (SHS) and third-hand smoke (THS), together referred to as tobacco smoke exposure (TSE) [3]. In addition to the health risks of TSE – which in children include lower respiratory infections, ear infections, asthma and sudden infant death syndrome (SIDS) [4], as well as delayed lung development and lifelong cardiovascular risks [5] - children of smoking parents are more likely to become smokers themselves [6, 7]. Despite improvements over the last decades in the number of families reporting smoke-free homes, many children are still exposed [8].

Assessments of children’s exposure to tobacco smoke often rely on parental report, which may be inaccurate or misclassify smoking exposure [9]. Parents often under-report their children’s exposure to tobacco smoke, as indicated by studies which compared biomarkers of exposure with parental report, or children’s own reports of exposure with their parents’ reports [10–13]. It was suggested that measurement flaws may underlie the under-reporting of exposure by parents [12]. Other researchers have suggested alternative explanations, including parental denial of exposure, which may be caused by social desirability bias, recall inaccuracy and misunderstanding of exposure [14].

Much research has investigated risk perceptions in the field of smoking, particularly regarding smoking around children. While some studies showed that the majority of parents considered that other people’s smoke could harm their child to some degree [15]; other investigators reported that parents who were smokers considered exposure of infants to tobacco smoke to be less dangerous than did non-smoking parents [16, 17]. However, parents’ perceptions of their children’s tobacco-related risks are based on how they understand the meaning of tobacco smoke exposure. A recent qualitative study [18] showed that people perceive tobacco exposure in different ways. These perceptions of exposure may influence parental risk perceptions and decision-making processes when it comes to smoking around their children. Some people consider exposure to occur only when tobacco smoke can be *seen* or *smelled;* while others have a much broader perception of what constitutes exposure. Some parents consider thirdhand smoke in their definition of exposure, while many do not.

In the context of children’s tobacco smoke exposure, researchers often ask parents whether or how often their child is exposed to cigarette smoke. People interpret these questions differently, thus giving differing responses for what objectively might be considered similar behaviour. A recent study found that people asked about their own SHS exposure interpret questions differently and recommended that survey questions should “consider alternative strategies for asking smokers questions about SHS” [19]. These differences in interpretation hinder comparison between subjects and across populations. Varying perceptions of exposure may explain parents’ subjective reports of their children’s exposure, their perception of their children’s risk, and ultimately their smoking behaviour around their children. Indeed a recent review of qualitative studies suggested a lack of understanding among parents of both SHS exposure and what constitutes a smoke-free home [20].

While there are existing measures of perceived exposure to tobacco smoke [21, 22], these assess one’s own perceptions of being exposed; there are currently no established tools to measure parents’ perceptions of children’s exposure to tobacco smoke. In order to fill this gap, we first conducted a qualitative study to learn how parents perceive TSE [18, 23] and from this developed a conceptual model to describe parental perceptions of TSE; and subsequently developed an instrument to assess PPE including rating the intensity of exposure in different circumstances. This paper describes the process of development and validation of the instrument, using an online survey and reports on the psychometric properties of this tool.

## Methods

**Fig. 1:**
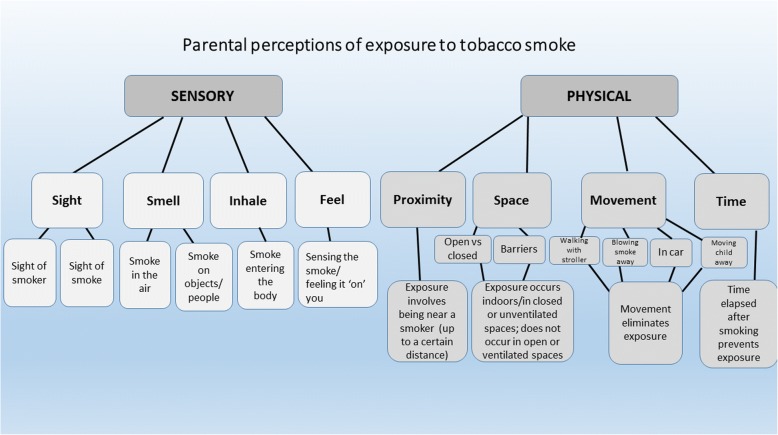
Conceptual model: Parental perceptions of children’s exposure to tobacco smoke. [Reprinted from Rosen et al. Parental Perceptions and Misconceptions of Child Tobacco Smoke Exposure, Nicotine & Tobacco Research 2017 doi: 10.1093/ntr/ntx169 by permission of Oxford University Press (licence no. 4399291196149)]

A questionnaire was constructed based on the results of a previous qualitative study conducted with 65 Israeli smoking parents, involving in-depth interviews. The analysis is reported in detail elsewhere [18]. Briefly, parents were asked to define exposure to tobacco smoke and from their responses a model was developed (Fig. 1). The analysis yielded two overall concepts regarding parental perceptions of exposure: (1) sensory perceptions (smell, sight) which parents rely on to determine whether exposure is occurring; and (2) the physical context, regarding proximity, space, movement, and time, whereby parents consider certain spaces as safe from exposure, certain distances as sufficient to prevent exposure, or certain amounts of time as sufficient to have elapsed between smoking and the presence of a child. Parents also expressed uncertainty about their knowledge on the matter. This model was then used to construct a questionnaire to quantify parental perceptions of exposure (PPE). Study items were developed to represent each of the arms of the model, using both pictures and vignettes of parents smoking around small children. (See Additional file 1 for full questionnaire). Items included indoor and outdoor smoking situations, barriers such as windows and doors, smoking in the car with windows open, and situations involving various distances between the smoker and child; and varying amounts of time between the end of smoking and the presence of a child; using sight and smell to detect exposure to smoke; and certainty regarding their answers. Respondents were asked to rate how they perceive the exposure of a hypothetical child in each given situation, with no relation to actual exposure of their own children.

Development of the questionnaire included content and face-validity, construct validity using factor analysis, reliability and internal consistency using test-retest reliability and Cronbach’s alpha correlation coefficients.

### Face validity

A preliminary version of the questionnaire was piloted with 22 parents in smoking families who were participants in a pilot study of Project Zero Exposure [24]. This initial version contained 10 photos and 10 descriptions of people smoking around children, to be rated according to the level of perceived exposure on a scale of 1 to 3. The purpose of using photos and text descriptions was to anchor the questions, so that each respondent would relate to the same unambiguous situation [25]. Preliminary testing showed variance in scores and indicated that respondents perceived different circumstances as constituting exposure to tobacco smoke at different levels.

The original version used available stock photos which matched some of the situations we wanted to represent but not all – it was therefore decided to create a new set of photographs using the same set of subjects in all of the different situations for unity, and to obtain higher resolution photos. We also changed the rating scale to a 7-point Likert scale to allow for greater variance and because 7-point scales have been recommended for risk perception questions [26]. The revised version of the questionnaire was piloted with a small group of 10 parents to assess feasibility and readability, ease and clarity of questions, formatting, response scales and pictures. Small changes were made to wording and picture quality following this initial pilot.

**Fig. 2:**
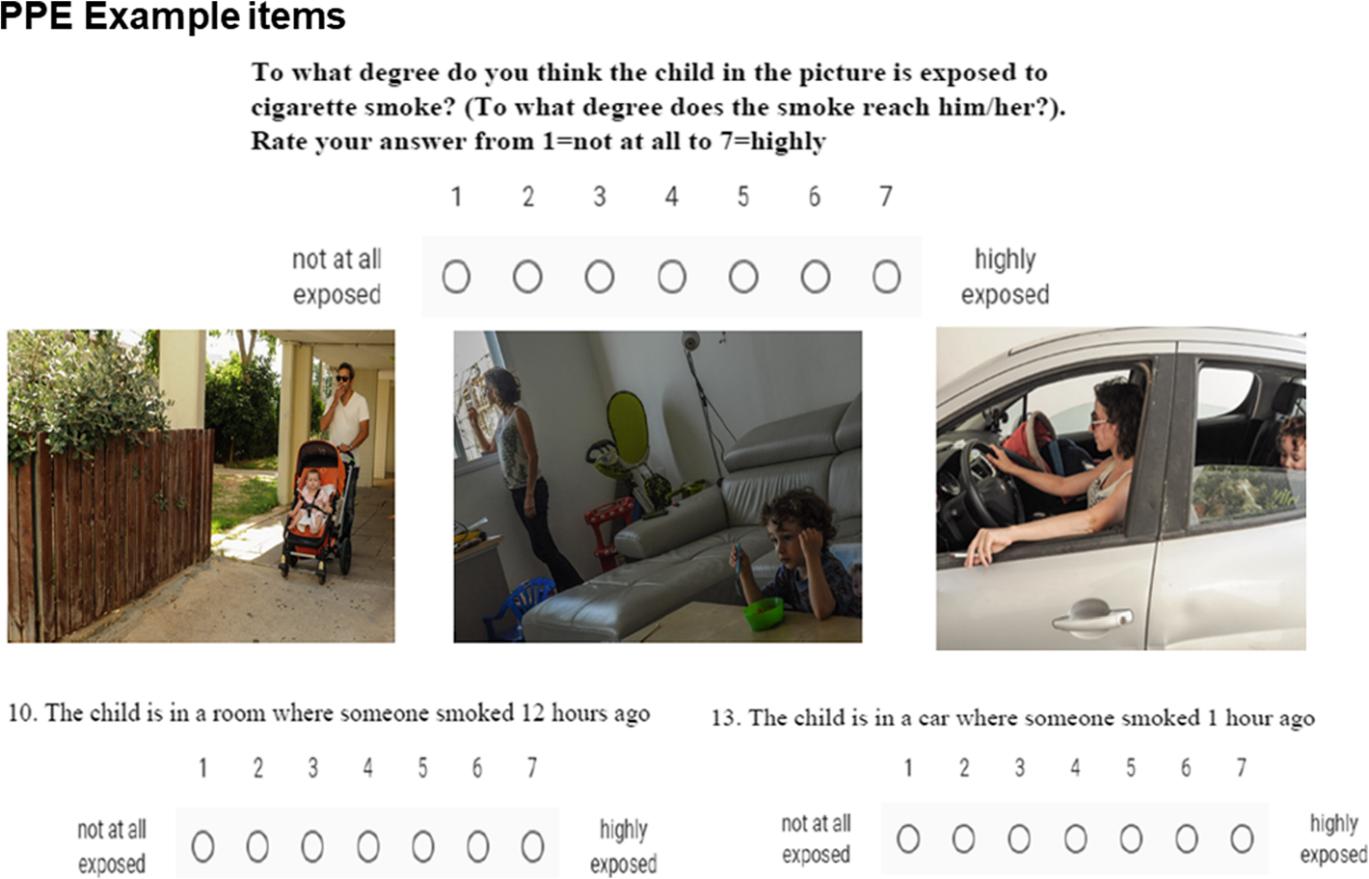
Example items from the PPE tool

The final version of the PPE questionnaire included a total of 24 items (see Fig. 2 for example items; and Additional file 1 for full questionnaire): 8 picture items, 9 text vignettes and 7 further questions regarding perceived knowledge. Participants were asked to score each item according to the level of exposure experienced by the child in the picture or described in the situation (“to what extent do you consider the child to be exposed to cigarette smoke? (to what extent does the smoke reach him?”). It was decided to add the explanation ‘to what extent does the smoke reach him’ in order to be specific and concrete and to ensure that respondents understood the question, in light of our previous findings that parents understand exposure in different ways [18]. All items were scored on 7 point Likert scales from 1 not at all exposed to 7 highly exposed. One text item asked participants to what extent they consider opening a window while smoking in the car to prevent exposure (from 1 not at all to 7 very much). Three items asked how long it takes for smoke to clear in the house and car; and how far smoke reaches outdoors – these three items were open-ended, and responses were classified into categories of 1–7 for each question [for time: 0–1 h/2-3 h/4–14 h/15–24 h/several days-week/months/years or never; for distance: 0-2 m/3-5 m/6-10 m/11-20 m/21-50 m/51-100 m/> 100 m]. Three further items assessed participants’ perceived knowledge and confidence in their responses (“do you consider that you have sufficient information on passive smoking?”, “how confident were you of your answers?”, “how difficult was it to answer the questionnaire?”), also scored on a 7 point Likert scale. PPE sum was obtained by summing all the item scores; PPE mean score was obtained by dividing the total score by the number of items answered.

### Construct validity

Factor Analysis was conducted to obtain construct validity.

### Research setting

The validation study was conducted in Israel. Current smoking rates are around 20.5% in Israeli adults when last surveyed in 2017: 25.9% in men and 15.3% in women; 22.9% in the Arab population, 20.0% in the Jewish population [27].

### Sample

To obtain a sample for factor analysis, the questionnaire was published in Hebrew (mother tongue of 80% of the Israeli population) on social media parenting groups from diverse geographical locations around Israel in August 2016, inviting parents with children up to age 18 to take part. There were no exclusion criteria and parents of all ethnicities were eligible to participate. According to a survey conducted by the Central Bureau of Statistics approximately 89% of Israeli adults in the target age group of parents to young children (ages 25–44) regularly use the Internet [28]. Participation was anonymous with the option to provide a contact email address. Consent was obtained at the start of the questionnaire. Data on age and sex of responding parent, mother tongue, number of children, age group of children, smoking status and SES (below average, average, above average) were collected.

We aimed for a sample of 200 based on the suggested use of at least five participants per variable [29]. There were a total of 224 responses – 3 were excluded due to having children over age 18 only; and 1 was excluded for not ticking the ‘agree to participate in the study’ box. This left 220 complete responses to be included in the analysis.

### Criterion validity

In the absence of a similar scale for comparison, PPE scores were compared with parental perceptions of risk (PPR) for cross-validation - a composite score made up of a total of 17 items which followed the same format as the PPE to enable comparison and validation: 8 pictures and 9 text items, scored on 7 point Likert scales from 1 = no risk to 7 = high risk, posing the question: “What do you consider the extent of the health risk to the child pictured/described” - a typical risk perception question, though referring here to a hypothetical situation. In addition, smoking status of the parents - the ultimate behavioural criterion – was also used to validate our new perceived exposure measure.

### Statistical analyses

Factor analysis was used to enable the clustering of items into a small number of factors [30]. Maximum likelihood was chosen as the method of extraction. As this is a new construct with no prior literature about how many factors the scale measures, factors with eigenvalues greater than 1 were retained, rather than specifying the required eigenvalue. An oblique rotation was used as we expected that factors would be correlated with each other. Factor loadings were ordered by size and those less than 0.10 were excluded from the output. Logistic regression was used to determine the power of PPE score to discriminate smokers from non-smokers. ANOVA was used to compare mean PPE score and sub-scores by demographic factors. Spearman’s correlation was used to assess the relationship between PPE and continuous variables.

### Ethics

Ethical approval of the study protocol was obtained from Tel Aviv University’s Institutional Review Board. Informed consent was obtained from participants, who had to provide their agreement to participate in the research before completing the questionnaire, which was anonymous.

## Results

### Participants

Participants included in the analysis were 220 Israeli parents of children up to age 18. Sample characteristics are presented in Table 1. Respondents were mostly mothers (86%), in their thirties (IQR 31–38 years), with young children (63% with children up to age 5; 83% up to age 10), with mostly Hebrew mother tongue. Almost half of respondents (47.2%) were either smokers or ex-smokers. The majority of respondents classified themselves as of average or above average SES (83.7%).

**Table 1:** Description of sample

	Mean (SD); n	Range	IQR
Age	34.79 (5.58); 211	21–55	31–38
Number of Children	1.97 (0.89); 219	1–4	1–3
Number of cigarettes/day (answering parent)	10.41 (6.57); 73	1–30	5–15
Number of cigarettes/day (partner)	11.78 (7.86); 64	1–30	5–15
Sum number of cigarettes/day (mother + father)	15.54 (11.34); 96	2–55	6–20
		N	%
Gender	Fathers	30	13.6%
	Mothers	190	86.4%
Smoking status	Smokers	72	32.7%
	Non-smokers	116	52.7%
	Ex-smokers	32	14.5%
Partner smoking status	Smoking partner	60	27.3%
	Ex-smoker partner	28	12.7%
SES	Below average	33	15%
	Average	106	48.2%
	Above average	78	35.5%
Mother tongue	Hebrew	187	83.1%
	Russian	16	7.1%
	Arabic	3	1.3%
	English	8	3.6%
	Other (not specified)	10	4.4%

Mean Number of cigarettes/day = mean of smoking respondents

#### Exploratory factor analysis

The Kaiser-Meyer-Olkin (KMO) measure of the sampling adequacy was 0.83 and Bartlett’s sphericity test was significant at *p* < .001, confirming suitability for performing exploratory factor analysis. Factor analysis revealed 7 factors for PPE. The first 7 factors (out of 24 items) were considered meaningful with eigenvalues greater than 1 – the model explained a cumulative total variance of 78.83%. However the rotation with this model failed – even after 30 iterations. The “open window” question, which seemed the least connected to the other items, was therefore removed, and the analysis redone (See [29]: unrelated items which do not belong together should be deleted). Removing the “open window” question from Factor 3 (Knowledge) improved its alpha coefficient from 0.609 to 0.691.

Running the second factor analysis on 23 items yielded 6 factors with eigenvalues greater than 1. This model explained a cumulative total variance of 76.3%. The factors are presented in Table 2.

**Table 2:** Factor labels and Cronbach scores

Factor	Label	Description	No of items (loadings range)	Cronbach’s Alpha
1	Second-hand exposure	Exposure by active smoking in car, home, outdoors	9 (.627–.894)	0.934
2	Third-hand exposure	Exposure after someone has smoked in car or home at various times	5 (.702–.928)	0.921
3	Perceived knowledge/certainty	Perceived knowledge, certainty and difficulty answering	3 (.717–.872)	0.690
4	Sensory exposure	Sight and smell	3 (−.706- -.847)	0.808
5	Time perceptions of exposure	Time taken for smoke to disperse after smoking	2 (.884–.919)	0.857
6	Space perceptions of exposure	Distance smoke travels	1 (.829)	–

Item specific statistics can be provided upon request

Item-total statistics were obtained for the 9 items of Factor 1, and for the 5 items of Factor 2, to determine if the factor should be condensed and any items removed for brevity of the scale. However, results showed that removal of any items would have reduced the alpha coefficient. Factor 6 (distance) had only one item but with a high loading; it was decided to add an extra question relating to this construct which was considered to be important to perceptions of exposure (‘how far does smoke reach outdoors on a windy day?’). The extra question was included in the revised questionnaire completed by an additional set of 80 parents. There was significant correlation between the two distance-related items of rho = .664 (*p* < .001).

#### PPE scores and range

Mean item scores ranged from 4.17 (SD = 1.67) for the text item “Being in a room where someone smoked 12 hours ago” to 6.66 (SD = 0.93) for the picture of a mother smoking in the kitchen near her child. Mean PPE score was 5.61 (SD = 1.02), range 1.94–7.0, *n* = 220 (out of a maximum of 7). Mean PPE sum was 95.22 (SD 17.4) range 33 to 119.

#### Test retest reliability

10% of respondents were asked to complete the questionnaire again a month later (only those who had voluntarily provided an email address could be approached) of which 13 participants complied (6% of the total sample). The subset was representative of the total sample with no significant differences in age, SES or smoking status. The correlation between total PPE score at T1 and T2 was *r* = 0.856, *p* < .001 demonstrating stability of responses and good reliability. Adequate test-retest reliabilities were also found for 5 of the 6 subscales (Factor 1: 0.824, *p* = 0.001; Factor 2: 0.800, p = 0.001; Factor 3: 0.609, *p* = 0.036; Factor 4: 0.778, *p* = 0.002; Factor 6: .673, *p* = .023). Factor 5 had poor reliability (0.420, *p* = 0.580). This can be attributed to the fact that it was based on open questions about time taken for smoke to disperse from various places, which produced a wide range of answers, later categorised for ease of classification; additionally there was a high rate of missing values for these questions. Factor analysis was performed again with these items excluded, resulting in 5 factors which explained a total variance of 73.6%. Due to the reduction of the total explained variance, it was decided to retain Factor 5, but to change the response format to closed categories for future use.

### Association between perceived exposure, perceived risk and demographic variables

**Fig. 3:**
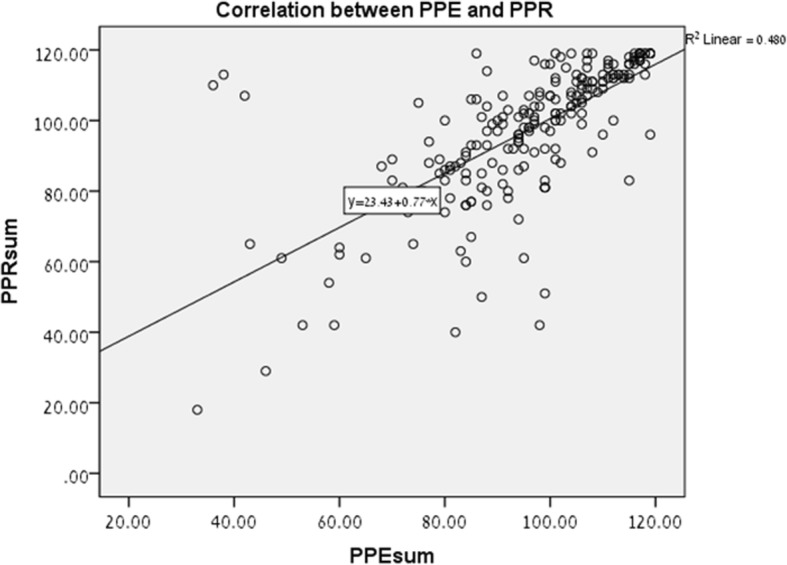
Scatterplot of the correlation between PPE and PPR

There was a high, significant correlation (Spearman) between PPE and perceptions of risk (PPR) sum scores (*r* = 0.766; *p* < 0.001) (Fig. 3). PPE Factor subscales and PPR total scores were also significantly correlated (see Table 3). Comparison between mean exposure and risk perceptions for specific items showed significantly lower risk scores and higher exposure rating, for example for smoking in the kitchen (PPE 6.66 vs PPR 6.38; paired t test *p* = .001).

**Table 3:** Spearman’s Correlations between PPE subscales and PPR score

	Factor 1 Secondhand exposure	Factor 2 Thirdhand exposure	Factor 3 Perceived knowledge	Factor 4 Sensory	Factor 5 Time	Factor 6 Distance	PPR score
Factor 1		0.534*	0.150	0.528*	0.439*	0.201*	0.613*
Factor 2			0.176*	0.596*	0.546*	0.073	0.646*
Factor 3				0.235*	0.166*	0.165*	0.262*
Factor 4					0.398*	0.139	0.877*
Factor 5						0.151	0.514*
Factor 6							0.165*

*signifies *p* ≤ 0.05

### Smokers versus non-smokers

Smokers had significantly lower PPE scores than non-smokers meaning that non-smokers had a broader definition of exposure and defined more situations as involving greater exposure, (*p* < 0.001). Interestingly ex-smokers had mean total scores closer to those of non-smokers (see Table 4) as demonstrated by post-hoc tests (Scheffe’s) which highlighted the significant difference between current smokers and non-smokers (*p* < .001), and between smokers and ex-smokers (*p* = .002), but not between ex-smokers and non-smokers (*p* = .771).

**Table 4:** Smokers versus non-smokers PPE scores

	Mean PPE sum (SD)	Mean PPE score	Factor 1	Factor 2	Factor 3	Factor 4	Factor 5	Factor 6
Smokers	85.58 (19.17) *n* = 72	5.03 (1.13) *n* = 72	5.09 (1.32) *n* = 72	5.09 (1.49) *n* = 72	4.62 (1.27) *n* = 71	4.86 (1.38) *n* = 71	3.23 (1.76) *n* = 54	3.35 (1.84) *n* = 49
Non-Smokers	100.41 (12.70) *n* = 116	5.91 (.75) *n* = 116	5.97 (.84) *n* = 116	5.84 (1.06) *n* = 116	4.41 (1.57) *n* = 115)	5.80 (1.10) *n* = 112	4.06 (1.80) *n* = 86	3.46 (1.74) *n* = 71
Ex-smokers	98.09 (19.38) *n* = 32	5.77 (1.14) *n* = 32	5.84 (1.21) *n* = 32	5.71 (1.38) *n* = 32	4.47 (1.43) *n* = 31	5.65 (1.28) *n* = 32	4.26 (1.64) *n* = 23	4.04 (1.77) *n* = 25
*p* value (ANOVA)	< 0.001	< 0.001	< 0.001	< 0.001	.625	< 0.001	.012	.267

*PPE* parental perceptions of exposure, *PPE sum* sum of all item scores, *PPE score* mean score, that is sum divided by the number of items answered

There were also significant differences between smokers and non-smokers on most subscales with non-smokers scoring consistently higher than smokers (see Table 4). We further found that a significant negative correlation between number of cigarettes smoked daily by parents and PPE scores, with heavier smokers giving lower ratings (*r* = −.243; *p* = .017).

A logistic regression was also conducted to determine the predictive power of PPE score on smoking status. Responding parent’s age, age of children and SES were included as covariates. A test of the full model against a constant model was statistically significant, indicating that the predictors as a set significantly distinguished between smokers and non-smokers, explaining 72.3% of variance in smoking status. According to the Wald test demographic factors were not significant predictors but PPE score was a significant predictor of smoking status (OR .494; *p* < 0.001). PPR was also entered into the equation but did not add significantly to predicting smoking status. A second logistic regression was conducted with PPE as the binary dependent variable (over or under median), as predicted by smoking status (current smoker or not) – this model was also significant (OR = .238, *p* < .001).

#### Mothers versus fathers

Mothers had a higher mean PPE sum than did fathers (96.4 ± 16.9 mothers vs 87.7 ± 18.9 fathers; *p* = 0.011; t test). Significant differences between mothers and fathers were also seen for Factors 1 (second-hand exposure) and 2 (third-hand exposure) but not for the remaining factors.

#### Age

Children’s age was negatively correlated with PPE sum (rho = − 0.135, *p* = 0.049, *n* = 214). Parents with the youngest children had significantly higher total scores (mean 100.4 ± 12.5; age 0–2) than those with older children (mean 90.4 ± 19.3; age 0–10) (*p* = 0.001). Parental age did not correlate with PPE.

## Discussion

We sought to create a valid instrument to measure parental perceptions of exposure (PPE) because of its likely importance in how parents understand exposure and in parental smoking behaviour around children. The developed instrument, based on prior qualitative work and a conceptual model [18], was tested in a sample of Israeli parents and found to be reliable and valid. Scores showed adequate variances relative to the theoretical ranges and all factors showed satisfactory internal consistency. A strong correlation was found between parental perceptions of children’s exposure and perceptions of risk to the child’s health. It is reasonable to assume that exposure perceptions precede risk perceptions, a proposition with theoretical and practical implications. If true, targeting exposure perceptions in intervention programs may lead to increased risk perceptions and ultimately changes in smoking behaviour. While exposure and risk perception scores were highly correlated, they often differed; that is, exposure is not always perceived as risky. Comparison of item ratings indicate for example, that while 80.9% gave smoking in the kitchen the top exposure rating, only 68.6% gave it the top risk score, implying that parents think differently about exposure and risk.

Exposure perceptions were lower among smokers compared to non-smokers – smokers perceived children as being less exposed to smoke in various situations, while non-smokers rated as higher the extent to which smoke reaches children when there is a smoker in the vicinity. This may be partly connected to sensory deficits of smokers. As we discovered in the qualitative stage of the study [18], people tend to rely on the smell or sight of smoke to determine whether exposure is occurring. However, research has shown that smokers are less sensitive to the smell of smoke and that regular exposure to strong smells raises the threshold of detection [31, 32]. Additionally, the differences between smokers and non-smokers in perceptions of exposure, whether preceding or concurrent with risk perceptions, may be interpreted as defensive responses or cognitive dissonance aimed to justify smoking and protect self-image and self-esteem [33]. These findings reflect reports of lower risk perceptions of passive smoking exhibited by smokers compared to non-smokers [16, 17]. A recent study of risk perceptions asked about the risk of smoking around a child in various circumstances (indoors/outdoors/in the next room) and found significant differences between smokers and non-smokers [34]. The relationship between cognitive dissonance, sensory perceptions and perceptions of exposure in smokers and non-smokers may require further research.

It was interesting to note that ex-smokers’ scores were more similar to non-smokers’ than to smokers’ exposure perceptions. It may be that increases in PPE -associated with increased risk perceptions - motivate and precede quitting smoking, as predicted by most social-cognitive models of health behaviour change [35]. Alternatively, it is possible that due to a need to reduce cognitive dissonance, beliefs about smoking change systematically with changes in smoking status. Due to the cross-sectional design of our study we cannot determine a causal direction between perceived exposure and smoking status, a question for further longitudinal research.

In relation to the model developed from the qualitative study, several theoretical elements directly translated into factors within the PPE. These include the sensory, time, and space/proximity factors. The time sub-scale is especially important in discriminating between second- and third-hand exposure, and also for defining third-hand exposure – how long ago did smoking occur and how does this impact perceived exposure?

Parents generally considered indoor smoking to involve more exposure than outdoor smoking [18]. However it was interesting to note that while 80.9% of respondents gave smoking in the kitchen near the child the top exposure rating; only 22.7% rated smoking on a closed balcony with the highest exposure score. This supports findings from a qualitative study that parents often do not consider a closed balcony to be ‘indoors’ or part of the house [18]. The factor related to perceived knowledge directly corresponds with themes of certainty and uncertainty about exposure, commonly raised among parents in the initial qualitative study. Interestingly, the perceived knowledge sub-scale was less correlated with the other sub-scales and with risk perceptions compared to the other factors. This corresponds with previous findings showing a weak association between factual understanding of smoking risks and self-evaluations of that understanding [36].

While several measures exist to assess perceptions of exposure [21, 22], these look at individuals’ perceptions of their own exposure to tobacco smoke, usually frequency of exposure, or number of smokers in the family in order to assess their own level of exposure. The crucial difference here is parents’ perceptions of children’s exposure, and understanding how someone else is affected by one’s smoking in different circumstances. The novelty of the PPE questionnaire is that it assesses how parents rate exposure in different circumstances, whereas other tools ask if exposure occurs in a dichotomous way or rate perceived frequency of exposure. Furthermore the PPE uses visual and textual vignettes as anchors to assess perceptions of exposure [25], so that respondents are relating to identical situations regarding space, distance and time as they affect exposure of children.

Understanding parental perceptions of tobacco smoke exposure may shed some light on discrepancies noted in the literature between self-reported and objective measures of TSE [10, 11]. For example, the National Health and Nutrition Examination Survey found objective cotinine measures to show much higher exposure in children compared to self-reports [11]. Similarly, in a sample of US smokers with either healthy children or children with asthma, biochemical assessment showed similar levels of exposure between groups, but parents of children with asthma reported lower SHS exposure than did parents of healthy children [10]. The under-reporting of exposure by parents was explained by either questionnaire design or denial of exposure, which may involve social desirability bias, recall inaccuracy and misunderstanding of exposure [14]. We suggest that variability in exposure perceptions may help explain different reporting outcomes – a hypothesis that we plan to test in the next stage of our research.

### Study limitations and strengths

One limitation of an anonymous internet survey is that we cannot know if people respond truthfully. However, studies have shown that especially for sensitive issues, people may be more likely to tell the truth in an anonymous survey compared to a confidential one [37]. The characteristics of the validation sample represent the typical user of parents’ groups on social media. Such parents of young children were highly represented in the survey, but parents from lower socioeconomic groups or those with poor digital literacy may have been under-represented. Indeed certain sectors of society were under-represented such as Arabs and ultra-Orthodox Jews who have lower use of Internet in general and social media in particular,and/or with non-Hebrew mother tongue. The tool is currently available in Hebrew and English, and may be translated for validation in other populations and cultures. While this initial validation was carried out with Israeli parents, perceptions of exposure seem to be based on sensory perceptions [18]; these perceptions are likely to be similar across countries and cultures. It is likely that the problem of perception of tobacco smoke exposure is not restricted to a single population and affects many populations, however this remains to be tested and validated in other groups. As in most surveys, participation was voluntary and may not reflect the non-responders. Because it was an internet-based survey, we were unable to obtain a response rate. Test-retest was performed on a small subset that was available (had provided email addresses voluntarily and could therefore be contacted again) – though there were no significant differences with the main sample, a larger subset would have been preferable. Since the PPE is a novel construct, there is no ‘gold standard’ to compare our results to; in order to overcome this limitation to the extent possible we compared perceptions of exposure and risk; and compared with the behavioural outcome of smoking status. The cross sectional design of the validation study precludes determining the directional causal effect between smoking status and PPE. A further longitudinal study and intervention studies are desirable to confirm this hypothesis. Future research may also examine the relationship between perceptions of exposure and biochemical measures of exposure to tobacco smoke.

## Conclusions

Results provide supporting evidence for the PPE as a reliable and valid construct, allowing quantification of parents’ perceptions of children’s exposure to tobacco smoke along 6 specific dimensions: second-hand and third-hand exposure, perceived knowledge and certainty, sensory, time and distance dimensions. Smoking parents were found to perceive exposure less frequently than non-smoking parents. Furthermore, PPE score was able to discriminate smokers from non-smokers. It would be important to know whether change in parental perceptions of exposure precedes or follows quitting smoking and other changes in parental smoking behaviour. PPE should be explored in further population groups to confirm these findings, which provide an innovative starting point for a new line of research. The PPE tool can help us understand parents’ smoking behaviour around their children and may be used in further research to investigate the relationship between perceptions, smoking behaviour and actual and reported exposure; and to determine whether and which parental perceptions of exposure can be altered by intervention. Misunderstanding of exposure to tobacco smoke not only hampers accurate assessment of exposure, but more importantly, perpetuates the exposure of a large percentage of the world’s children. Understanding how parents perceive exposure can help us tailor the information we provide to them, correct misconceptions, raise awareness of exposure in various circumstances and help parents better protect their children.

## Additional file

Additional file 1: Parental Perceptions of Exposure questionnaire. The full questionnaire is presented here complete with pictures (PDF 912 kb)

## Abbreviations

PPE: Parental perceptions of exposure
PPR: Parental perceptions of risk
SHS: Secondhand smoke
THS: Thirdhand smoke
TSE: Tobacco smoke exposure
