# Supplementary material for: Parental perceptions of children’s exposure to tobacco smoke: development and validation of a new measure
Source: BMC Public Health. 2018 Aug 20;18:1031. doi: 10.1186/s12889-018-5928-1 (PMC6102809; doi:10.1186/s12889-018-5928-1)
Supplement: Supplementary file 1 — Parental Perceptions of Exposure questionnaire. The full questionnaire is presented here complete with pictures (PDF 912 kb) [file 12889_2018_5928_MOESM1_ESM.pdf]

Appendix 1: Parental perceptions of exposure questionnaire (English translation)

A. **In this questionnaire you will be presented with various situations showing smokers and children.**
   To what degree do you think the child in the picture is exposed to cigarette smoke? (To what degree does the smoke reach him/her?).
   Rate your answer from 1=not at all to 7=highly

	1	2	3	4	5	6	7	
not at all exposed	O	O	O	O	O	O	O	highly exposed

1. 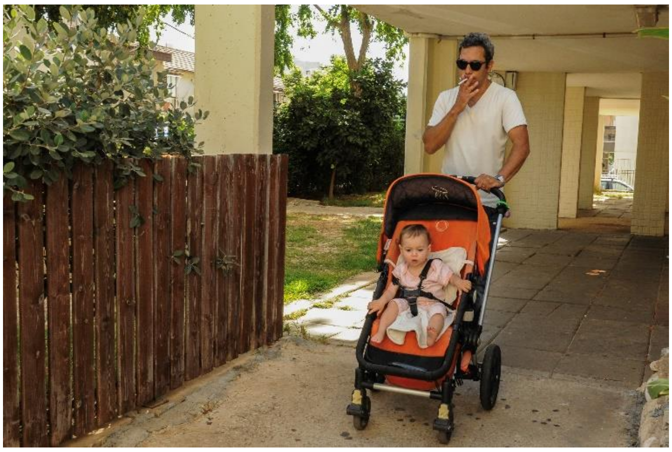

	1	2	3	4	5	6	7	
not at all exposed	O	O	O	O	O	O	O	highly exposed

2.

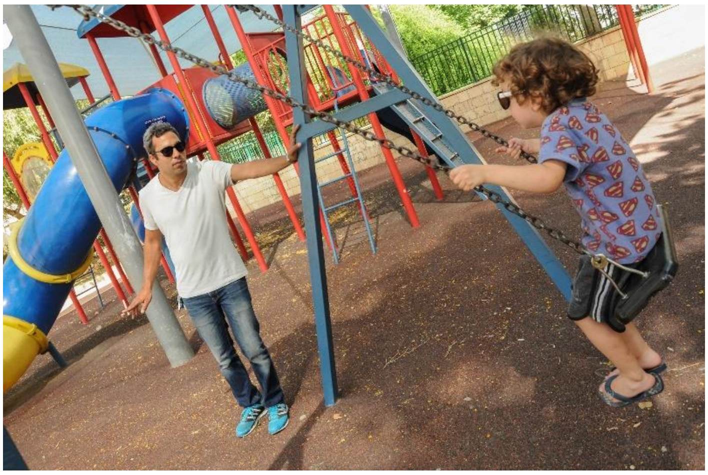

1	2	3	4	5	6	7	
not at all exposed	○	○	○	○	○	○	○	highly exposed

3. 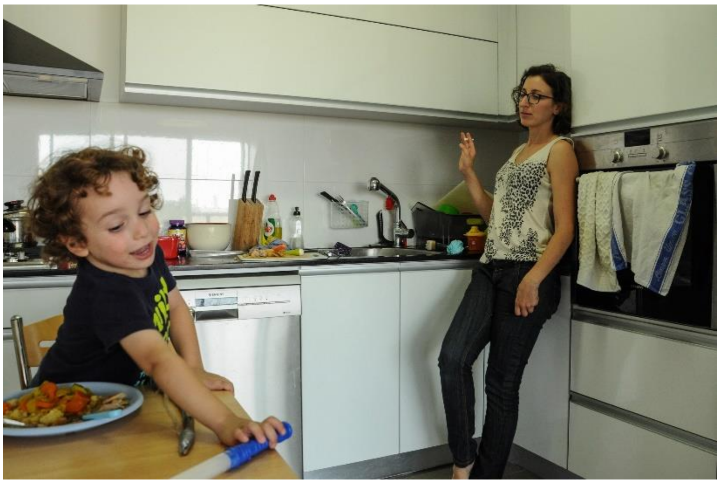

1	2	3	4	5	6	7	
not at all exposed	○	○	○	○	○	○	○	highly exposed

4. 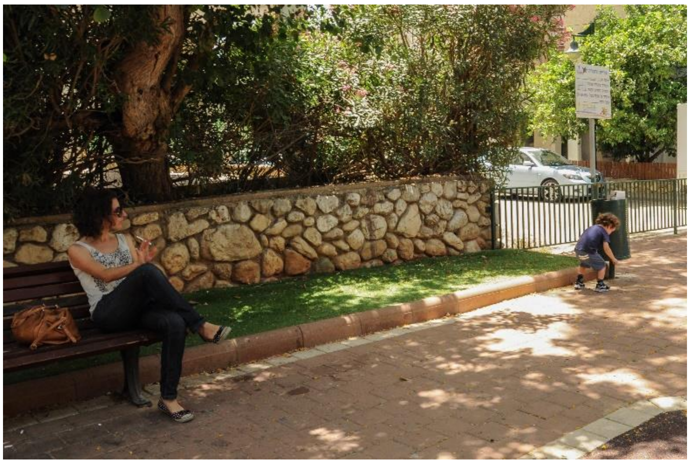

	1	2	3	4	5	6	7	
not at all exposed	O	O	O	O	O	O	O	highly exposed

5. 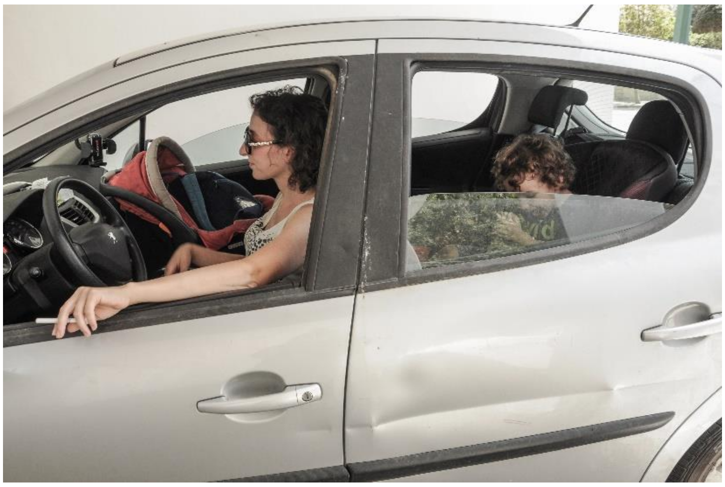

	1	2	3	4	5	6	7	
not at all exposed	O	O	O	O	O	O	O	highly exposed

6. 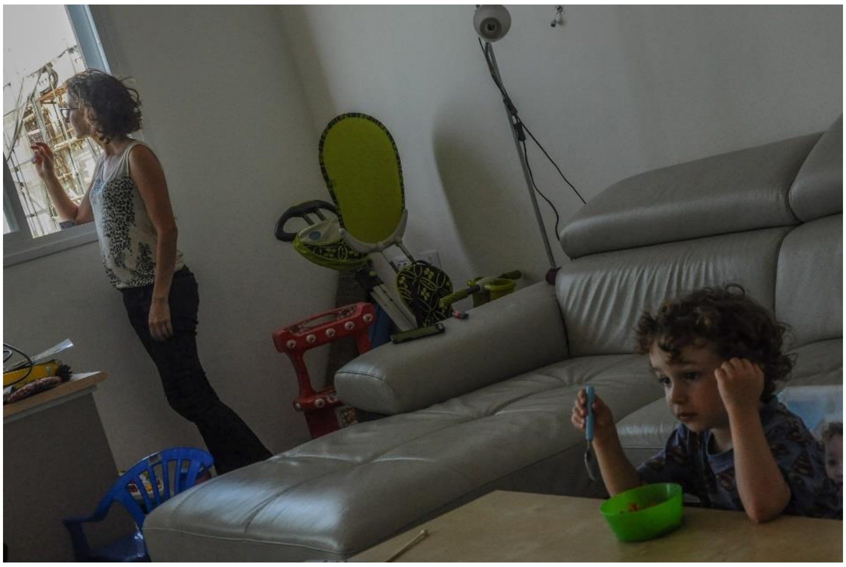

	1	2	3	4	5	6	7	
not at all exposed	O	O	O	O	O	O	O	highly exposed

7. 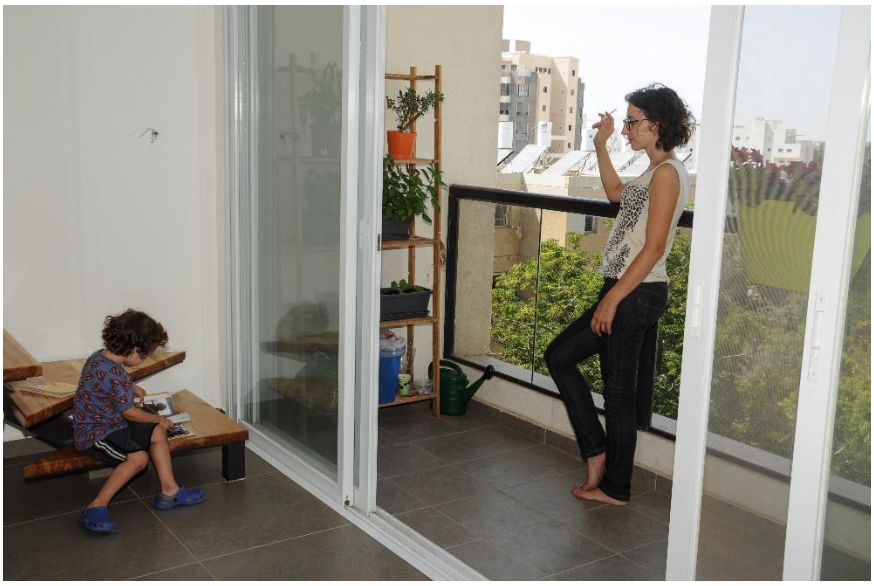

	1	2	3	4	5	6	7	
not at all exposed	O	O	O	O	O	O	O	highly exposed

8. 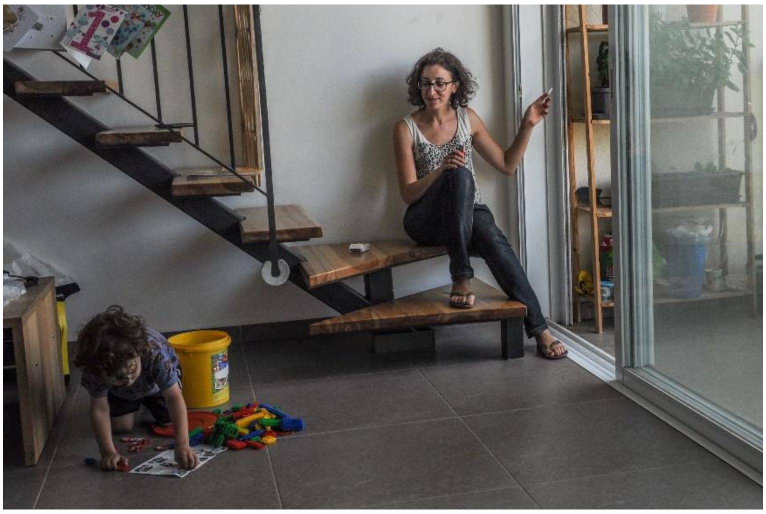

	1	2	3	4	5	6	7	
not at all exposed	O	O	O	O	O	O	O	highly exposed

B. In the following questions situations will be described without pictures. Please rate to what degree you think the child described is exposed to cigarette smoke? (To what degree does the smoke reach him/her?)

9. The child is in the kitchen, someone is smoking on the adjacent service balcony

	1	2	3	4	5	6	7	
not at all exposed	O	O	O	O	O	O	O	highly exposed

10. The child is in a room where someone smoked 12 hours ago

	1	2	3	4	5	6	7	
not at all exposed	O	O	O	O	O	O	O	highly exposed

11. The child is in a room where someone smoked 2 hours ago

	1	2	3	4	5	6	7	
not at all exposed	O	O	O	O	O	O	O	highly exposed

12. The child is in a room where someone smoked 30 minutes ago

	1	2	3	4	5	6	7	
not at all exposed	O	O	O	O	O	O	O	highly exposed

13. The child is in a car where someone smoked 1 hour ago

	1	2	3	4	5	6	7	
not at all exposed	O	O	O	O	O	O	O	highly exposed

14. The child is in a car where someone smoked 20 minutes ago

	1	2	3	4	5	6	7	
not at all exposed	O	O	O	O	O	O	O	highly exposed

15. The child is in the playground and sees his mother smoking but does not smell smoke

	1	2	3	4	5	6	7	
not at all exposed	O	O	O	O	O	O	O	highly exposed

16. The child is in the playground and smells cigarette smoke but does not see the smoker

	1	2	3	4	5	6	7	
not at all exposed	O	O	O	O	O	O	O	highly exposed

17. The child is in the playground and sees his mother smoking and can smell the smoke

	1	2	3	4	5	6	7	
not at all exposed	○	○	○	○	○	○	○	highly exposed

C. Next you will be presented with the same pictures, this time you will be asked to rate the health risk to the child in the described or pictured situation:

	1	2	3	4	5	6	7	
no risk	○	○	○	○	○	○	○	high risk

18. 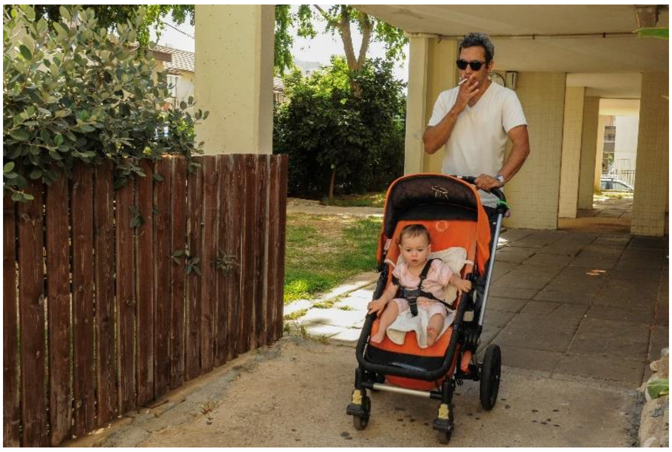

	1	2	3	4	5	6	7	
no risk	○	○	○	○	○	○	○	high risk

19. 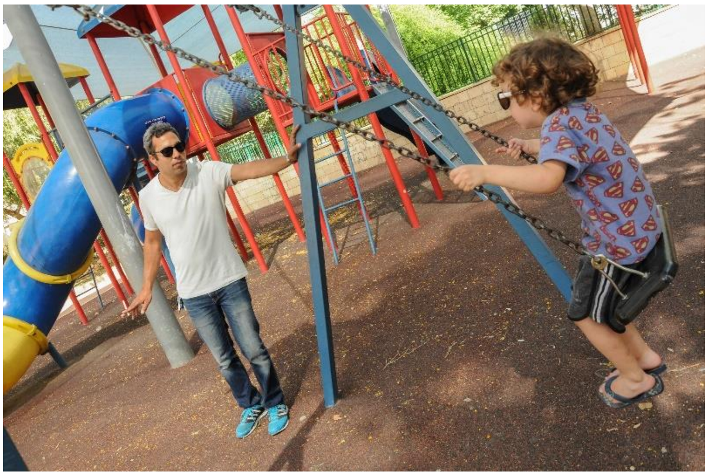

	1	2	3	4	5	6	7	
no risk	O	O	O	O	O	O	O	high risk

20. 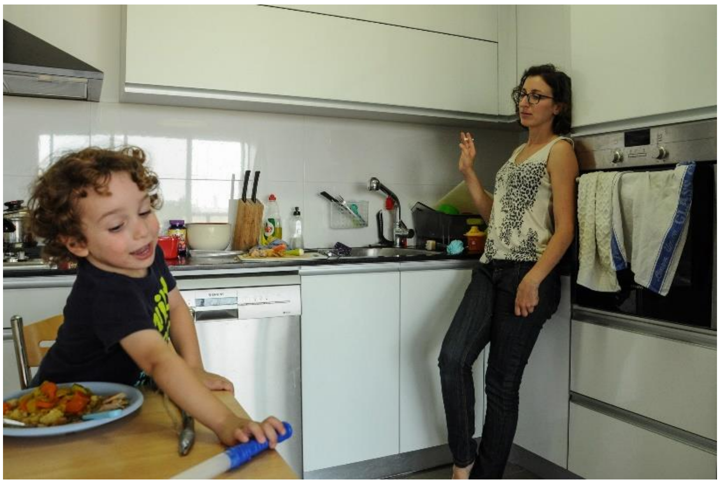

	1	2	3	4	5	6	7	
no risk	O	O	O	O	O	O	O	high risk

21. 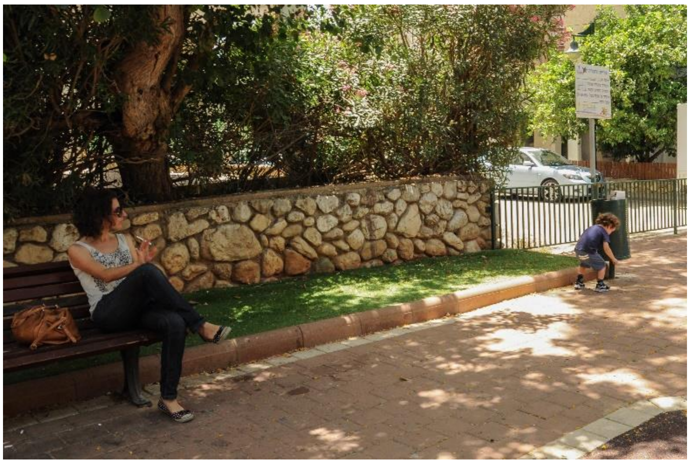

	1	2	3	4	5	6	7	
no risk	○	○	○	○	○	○	○	high risk

22.

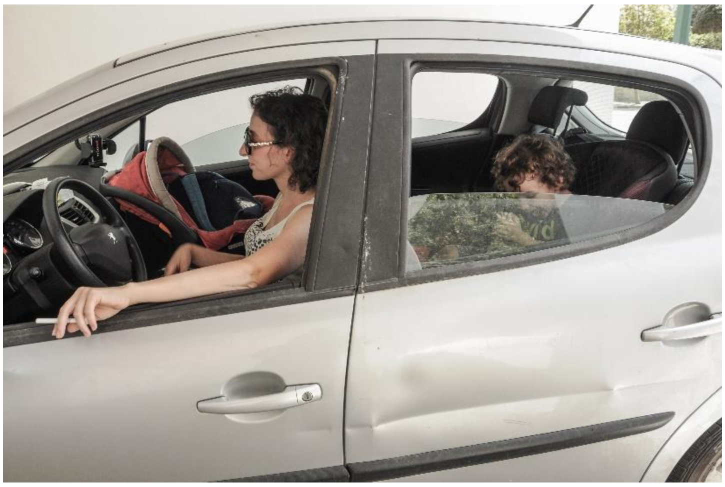

	1	2	3	4	5	6	7	
no risk	○	○	○	○	○	○	○	high risk

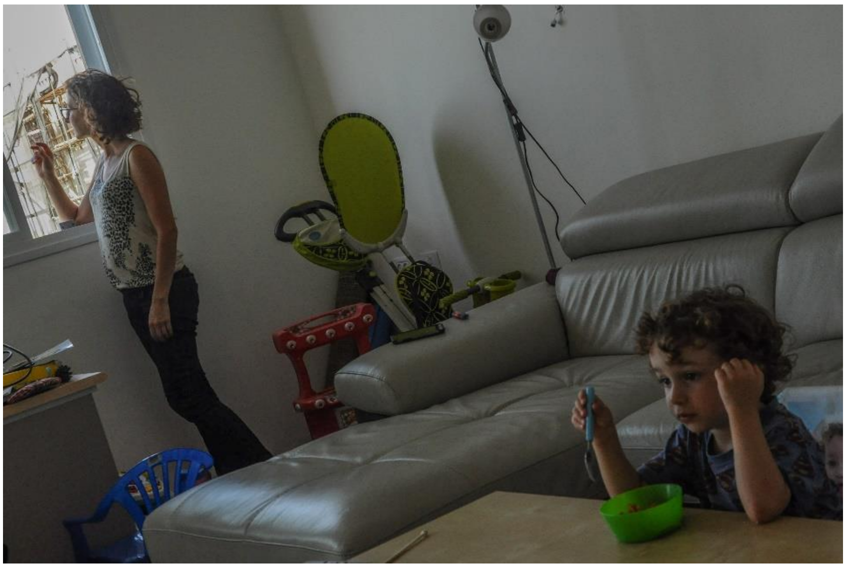

23.

	1	2	3	4	5	6	7	
no risk	○	○	○	○	○	○	○	high risk

24. 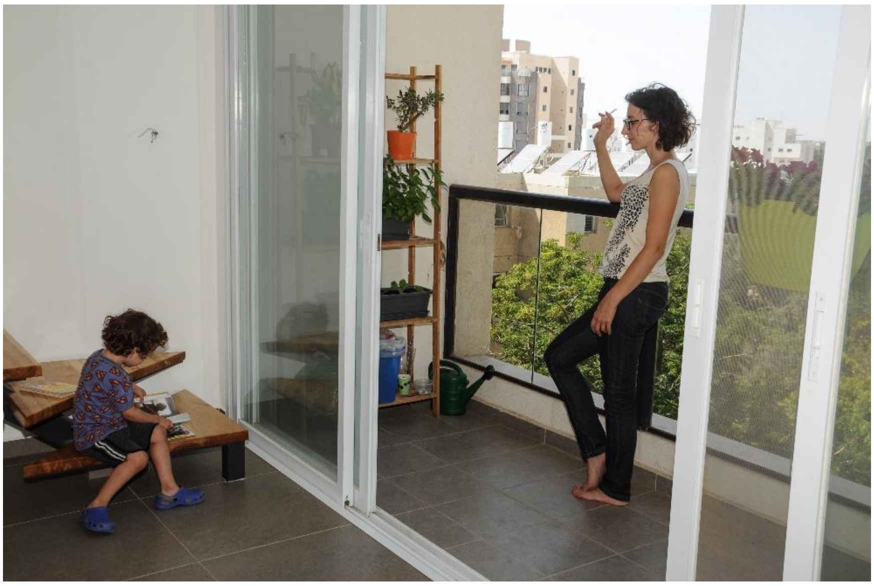

	1	2	3	4	5	6	7	
no risk	○	○	○	○	○	○	○	high risk

25. 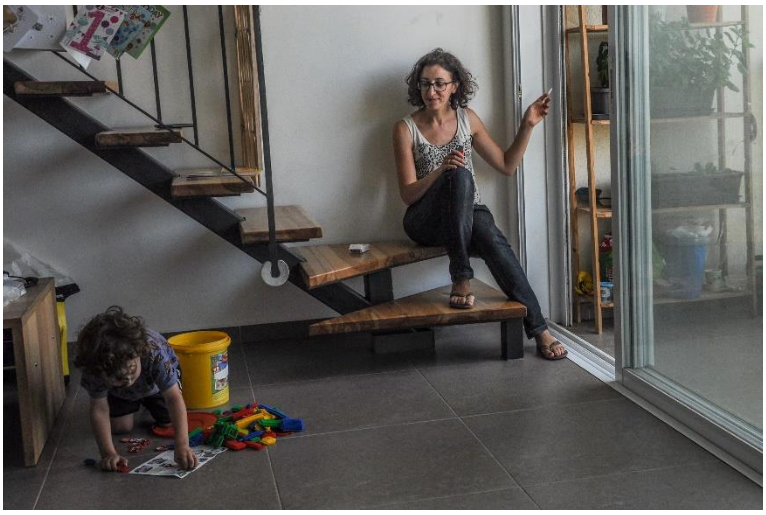

	1	2	3	4	5	6	7	
no risk	○	○	○	○	○	○	○	high risk

26. The child is in the kitchen, someone is smoking on the adjacent service balcony

	1	2	3	4	5	6	7	
no risk	○	○	○	○	○	○	○	high risk

27. The child is in a room where someone smoked 12 hours ago

	1	2	3	4	5	6	7	
no risk	○	○	○	○	○	○	○	high risk

28. The child is in a room where someone smoked 2 hours ago

	1	2	3	4	5	6	7	
no risk	○	○	○	○	○	○	○	high risk

29. The child is in a room where someone smoked 30 minutes ago

	1	2	3	4	5	6	7	
no risk	○	○	○	○	○	○	○	high risk

30. The child is in a car where someone smoked 1 hour ago

	1	2	3	4	5	6	7	
no risk	○	○	○	○	○	○	○	high risk

31. The child is in a car where someone smoked 20 minutes ago

	1	2	3	4	5	6	7	
no risk	○	○	○	○	○	○	○	high risk

32. The child is in the playground and sees his mother smoking but does not smell smoke

	1	2	3	4	5	6	7	
no risk	○	○	○	○	○	○	○	high risk

33. The child is in the playground and smells cigarette smoke but does not see the smoker

	1	2	3	4	5	6	7	
no risk	○	○	○	○	○	○	○	high risk

34. The child is in the playground and sees his mother smoking and can smell the smoke

	1	2	3	4	5	6	7	
no risk	○	○	○	○	○	○	○	high risk

35. Do you consider opening a window while smoking in the car to prevent children's exposure to tobacco smoke?

	1	2	3	4	5	6	7	
not at all	○	○	○	○	○	○	○	very much

36. What proportion of tobacco smoke is invisible?
    20%
    50%
    80%

37. After smoking in the home, how long does it take for the home to be smoke-free?______________________________ (open response)

38. After smoking in the car, how long does it take for the car to be free of smoke? ______________________________ (open response)

39. Do you consider yourself to have sufficient information on the subject of passive smoking?

	1	2	3	4	5	6	7	
not at all	○	○	○	○	○	○	○	very much

40. How confident did you feel of your answers?

	1	2	3	4	5	6	7	
not at all	○	○	○	○	○	○	○	very much

41. Did you find it difficult to answer the questionnaire?

	1	2	3	4	5	6	7	
not at all	○	○	○	○	○	○	○	very much

Thank you for your participation!
